# Complicated labral injuries in female patients with developmental dysplasia of the hip are associated with localized stress concentration in the superior acetabulum

**DOI:** 10.1007/s00402-026-06452-8

**Published:** 2026-10-21

**Authors:** Yuki Ogawa, Tomohiro Shimizu, Yusuke Ohashi, Yutaro Sugawara, Daisuke Takahashi, Norimasa Iwasaki

**Affiliations:** Department of Orthopaedic Surgery, Hokkaido University Hospital, Sapporo, Japan

**Keywords:** Developmental dysplasia of the hip, Labral injury, Stress distribution, Computed tomography osteoabsorptometry (CT-OAM)

## Abstract

**Introduction:**

Developmental dysplasia of the hip (DDH) alters joint mechanics and increases the risk of labral injury, compromising hip stability and leading to abnormal stress distribution and cartilage degeneration. However, the relationship between labral injury severity and joint loading stress in DDH remains unclear. This study investigated the effect of labral injury severity on stress distribution in DDH patients using computed tomography osteoabsorptometry (CT-OAM) and finite element analysis.

**Materials and methods:**

We analyzed 54 hips from 45 female patients undergoing joint-preserving surgery for DDH. Labral injuries were classified as simple or complicated on radial MRI. Subchondral bone density distribution was evaluated with CT-OAM, while finite element analysis of pelvic and femoral bones with cartilage was performed under a single-leg stance to assess contact pressure and area. Statistical analyses examined associations between labral injury severity and stress distribution.

**Results:**

Superior injuries showed significantly increased high-density area (HDA) in the superior acetabulum, indicating localized stress concentration (simple injury vs. complicated injury, 64.4% vs. 80.3%, *p* < 0.001). However, finite element analysis revealed no significant differences in contact pressure or area (simple vs. complicated, 5.3 N/mm² vs. 6.2 N/mm², *p* = 0.538), which we interpret as showing that bony morphology alone could not explain the observed stress patterns. Instead, the discrepancy highlights the role of labral failure reflected in CT-derived subchondral bone density.

**Conclusion:**

Complicated labral injuries, especially superiorly, were associated with localized stress accumulation in DDH.

## Introduction

Developmental dysplasia of the hip (DDH) is a morphological abnormality characterized by shallow femoral head coverage, resulting in concentrated loading stress at the edge of the hip joint and subsequent labral damage [1, 40]. Finite element (FEM) analysis has demonstrated that in a normal hip joint, the labrum bears only 1–2% of the total applied load, whereas in DDH, this increases to 4–11% [15]. Athletes with DDH, such as ballet dancers and long-distance runners, are at an elevated risk of labral injury due to repetitive hip movements and increased mechanical stress on the joint [8, 20]. Labral injuries are frequently associated with DDH, and understanding their pathogenesis may improve the treatment and management of this condition.

DDH is a known risk factor for secondary hip osteoarthritis (OA). Labral injuries are commonly observed in DDH, with labral tears or degeneration reported in approximately 90% of symptomatic cases requiring surgical intervention [37]. Although labral injuries can occur throughout the joint circumference, they are most frequently observed in the anterior region in patients with DDH, followed by the superior region [30, 34]. However, the precise role of labral damage in the progression of OA remains unclear. Specifically, it is uncertain whether labral damage leads to abnormal stress distribution within the joint or whether abnormal stress distribution primarily contributes to labral damage.

Because of the inherent capsular laxity in patients with DDH prior to surgical intervention, arthroscopy may further exacerbate capsular instability [6, 43]. Therefore, careful patient selection is crucial, as certain patients may be at risk for poor postoperative outcomes [14, 44]. Although arthroscopic surgery is a minimally invasive technique, it is not a viable option for patients with severe DDH because it does not restore the missing bony stability. Several studies have reported the efficacy of labral repair in cases of borderline DDH, defined as a lateral center-edge angle of 20° to 25° [13], as well as the benefits of additional suturing during periacetabular osteotomy (PAO) [2, 11]. However, the effectiveness of intra-articular interventions for labral injury in patients with DDH remains controversial. Clarifying the extent to which the severity of labral injury contributes to the abnormal distribution of joint loads caused by instability may provide valuable insights.

Given that nearly all symptomatic patients with DDH exhibit acetabular injuries with a predilection for specific locations, we hypothesized that the type and location of labral injuries may influence loading stress distribution across the hip joint. Therefore, in this study, we aimed to investigate the relationship between labral injury characteristics and loading stress distribution in patients with DDH. In addition, because DDH is fundamentally a morphological abnormality of the acetabulum, we further hypothesized that bony morphology itself would also contribute to these relationships. To achieve this, we used CT-osteoabsorptometry (CT-OAM) to estimate the cumulative loading stress [41], finite element analysis (FEA) to assess the impact of bone morphology, and radial magnetic resonance imaging (MRI) to evaluate labral pathology.

## Methods

### Patients

This single-center cross-sectional study was approved by the ethics committee of our institution. To collect data on DDH, we selected patients who underwent PAO for DDH at our hospital between 2016 and 2024. Data from preoperative examinations were collected and analyzed. A total of 58 patients (81 hips) were initially identified. However, 13 cases lacked applicable radial MRI data, 10 cases lacked the plain CT data required for CT-OAM analysis were excluded. In our cohort, there were 45 female patients but only four male patients, representing four hips. Because of this marked imbalance, it was difficult to appropriately evaluate sex-related differences. Therefore, the analysis was limited to female patients. The final analysis included 45 patients (54 hips). Power analysis was performed to detect a 10% difference in the clinical score between patients with and without MRI-positive findings (labral tears), with a power of 80% and a significance level of 0.05 in the previous study. Thirty-three participants were required, and we were able to recruit a sufficient number of patients for this study. The mean age was 30.3 years (95%CI, 27.0–33.6), and the mean BMI was 21.7 kg/m² (95%CI, 20.7–22.7). In addition, the Japanese Orthopaedic Association Hip Disease Evaluation Questionnaire (JHEQ) Movement subscale was assessed to evaluate movement-related functional status [29]. 

### Radiological evaluation

The degree of hip joint degeneration was assessed using the Tönnis classification [24], while hip dysplasia severity was classified according to the Crowe classification [19]. Supine anterior‒posterior pelvic radiographs were obtained preoperatively. Preoperative supine anterior–posterior pelvic radiographs were obtained to measure the center-edge (CE) angle, acetabular head index (AHI) [16], and acetabular roof obliquity (ARO) [28]. Femoral head positioning on anterior-posterior radiographs was evaluated using the method described by Nozawa et al. [35]. In addition, the anterior center-edge angle (ACEA) was assessed on false-profile radiographs [25]. Femoral cam lesions were evaluated using anteroposterior, lateral, and Dunn radiographic views to confirm their presence and anatomical location. The interobserver variability in the radiological findings between the two observers was 0.77–0.84.

### CT-OAM

CT-OAM, a method developed by Müller-Gerbl et al. for assessing radiodensity variations in Hounsfield units (HUs) [31], was used to evaluate the subchondral bone density in vivo. The distribution of subchondral bone mineral density (BMD) correlates with the cumulative stress applied to the joint surface, providing insights into stress accumulation and abnormal loading patterns [32, 36]. Furthermore, cartilage degeneration has been reported to occur in regions corresponding to areas of high subchondral bone density [9]. 

A high-resolution helical CT scanner (Aquilion One/ViSION Edition; Toshiba Medical Systems, Japan) with a 512 × 512-pixel matrix was used to obtain axial images of the hip, which had been acquired for routine clinical care. Slice thickness and interval were set to 1 mm. Coronal and sagittal slices based on the anterior pelvic plane, along with three-dimensional (3D) bone models, were generated from axial CT data using commercial software (Ziocube^®^; Ziosoft, Inc., Tokyo, Japan). CT-OAM was performed to evaluate the subchondral BMD using non-commercial software (OsteoDens 4.0) developed at our institution [18, 41]. 

**Fig. 1:**
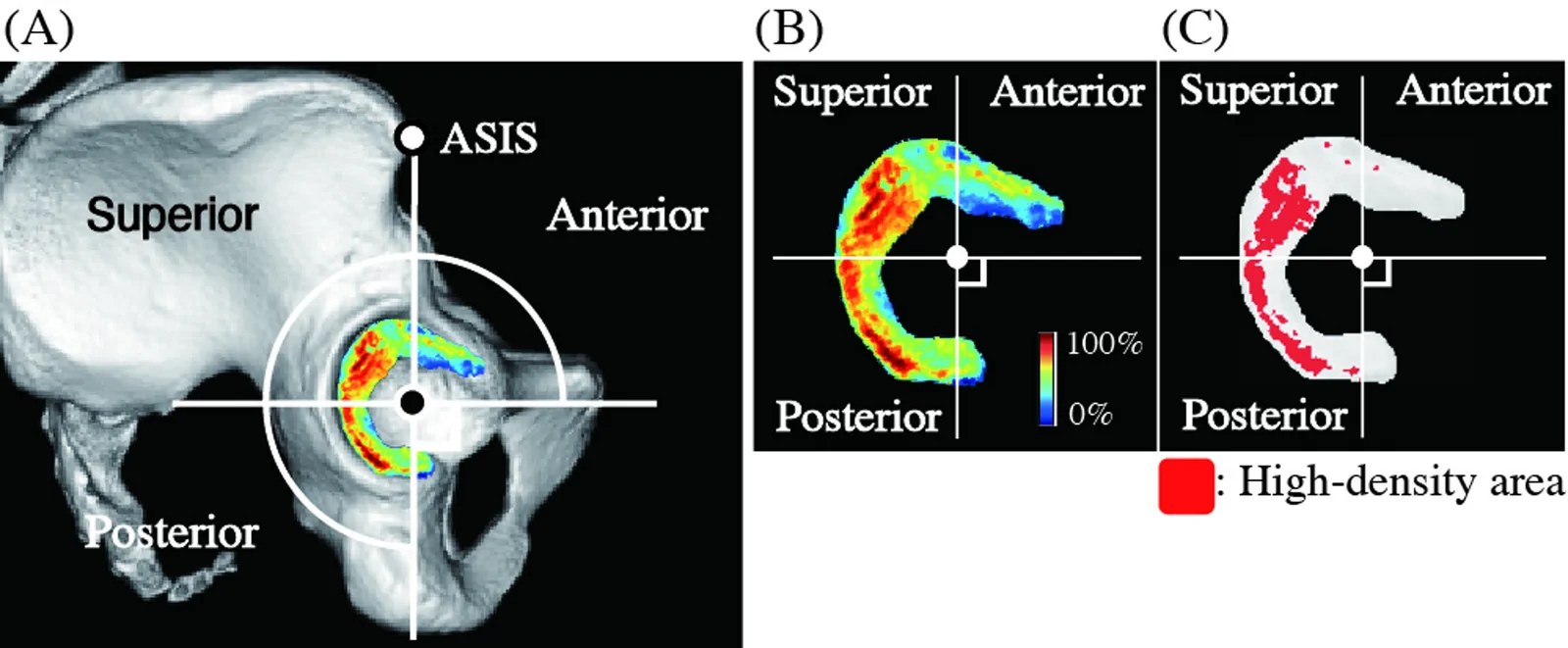
**A** Subregions of the hip joint. The baseline is defined as the line passing through the center of the acetabulum to the anterosuperior iliac spine (ASIS). The acetabulum is divided into three regions. **B** For quantitative analysis, the distribution pattern of subchondral bone density is visualized as a surface-mapping image using a color scale. **C** The high-density area (HDA), representing the top 20% of bone density values across the entire joint surface, is highlighted in red

In the coronal images, the region of interest was selected to include the acetabular subchondral bone layer in all the slices. Radiodensity was automatically measured in HUs at 1.0 mm intervals along the arc of the acetabulum, with one measurement per coronal slice. The measurements targeted a 2.5 mm region from the articular surface. To ensure consistency of HU values across patients, calibration phantoms scanned simultaneously with the patients were used as reference. Because the initial images included the acetabular floor and ligamentum teres attachment areas, the target region was manually refined to encompass only the acetabular subchondral bone, referencing individual slices and a 3D model. A two-dimensional (2D) density distribution map was generated by stacking slices, and the data were visualized using a color scale. The acetabulum was divided into three regions spanning 90° anteriorly to 180° posteriorly along a reference plane passing through the center of the acetabulum and the anterior superior iliac spine (Fig. 1A, B). Quantitative analysis focused on the high-density area (HDA), defined as the region containing the top 20% of the HU values across the articular surface (Fig. 1C). HDA percentage was calculated as the proportion of each region’s area occupied by HDA, indicating how the high-density region was distributed among the anterior, superior, and posterior regions. Two observers independently measured %HDA in these 10 hips; 30 subregions were anonymously measured twice at 3-month intervals. The intraclass correlation coefficient for intraobserver reliability was 0.92, and interobserver reproducibility was 0.89.

### MRI

Radial MRI was performed preoperatively using a Discovery MR750W scanner (3.0 T, GE Healthcare, Chicago, IL, USA). T2*-weighted images were obtained using a 2D gradient echo sequence (repetition time, 460 ms; echo time, 15 ms; flip angle, 30°).

**Fig. 2:**
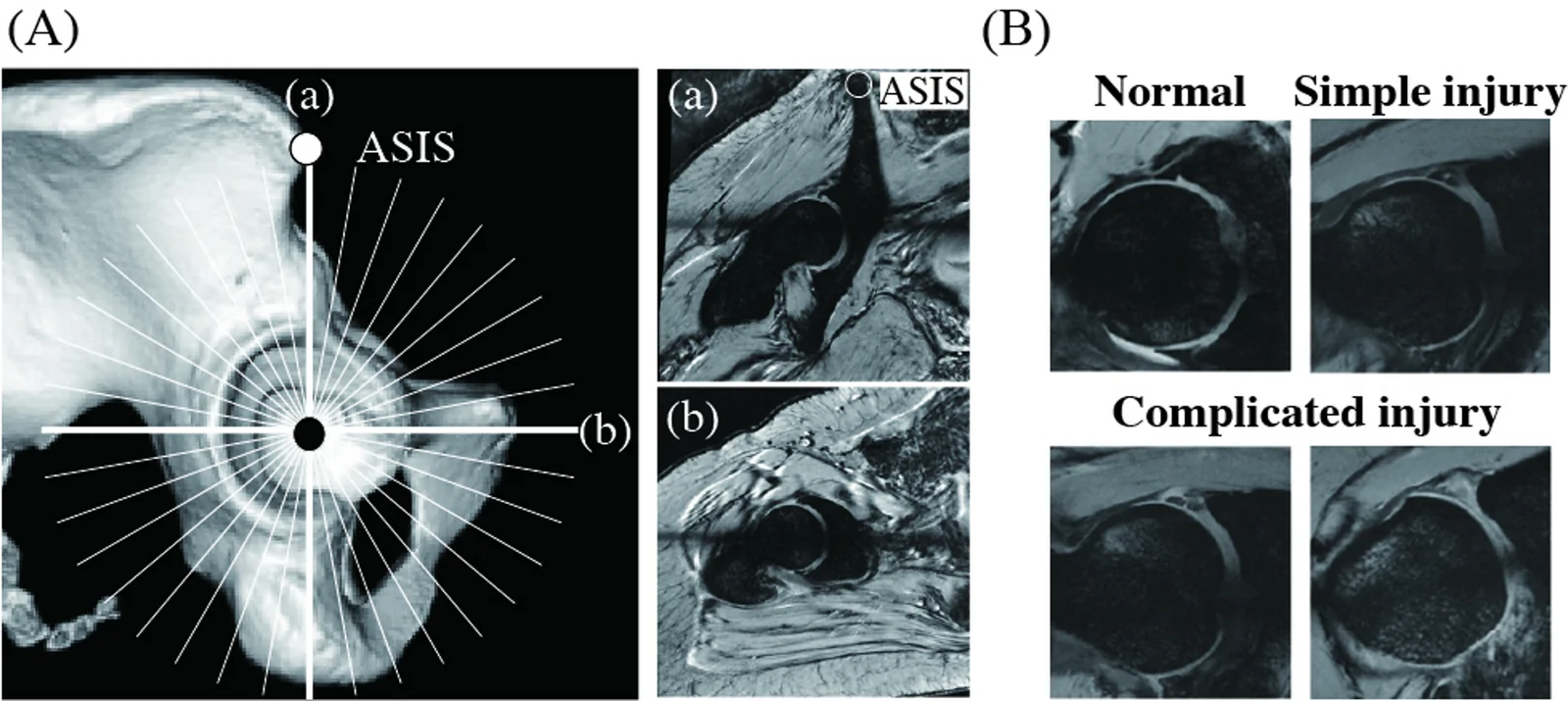
**A** Slice positions of radial imaging sections of the hip. The plane passing through the center of the acetabulum to the anterosuperior iliac spine (ASIS) served as a reference for automatically prescribing 18 radial sections at 10° intervals. Radial sections were acquired orthogonal to the acetabulum. The MRI images in (a) and (b) are shown on the right side of the panel. **B** Representative labral injuries categorized according to severity

The imaging planes were determined using the acetabular open view, which directly faces the acetabulum, after adjusting for acetabular anteversion and lateral inclination. Based on this localizer image, 18 radial slices were acquired at 10° intervals, passing through the center of the femoral head and perpendicular to the acetabular labrum (slice thickness, 4 mm) (Fig. 2A).

Labral lesions were categorized into two groups: simple injuries, defined as abnormal signals or simple tears; and complicated injuries, characterized by complex tears or macerations (Fig. 2B). An abnormal signal was defined as an intralabral signal change without contour disruption. For each MRI slice, a disruption of the labral contour confined to one site was considered a simple tear, while disruptions at multiple sites, consistent with multidirectional extension, were considered a complicated tear. Importantly, tears spanning several subregions but oriented in a single direction were regarded as simple injuries. Maceration was defined as a thickening, contour opacity, or signal changes involving the entire acetabulum. Labral injuries were assessed on each radial section, and for each of the three acetabular regions—anterior, superior, and posterior—the most severe finding among the radial sections was defined as the representative grade of labral injury for that region. Two observers independently classified a randomly selected dataset of 126 MRI slices from 7 hips. The interobserver agreement was 0.88. Intraobserver reliability was assessed by repeated evaluation performed more than 3 months apart, and the intraobserver agreement was 0.90.

### Finite-element (FE) analyses

**Fig. 3:**
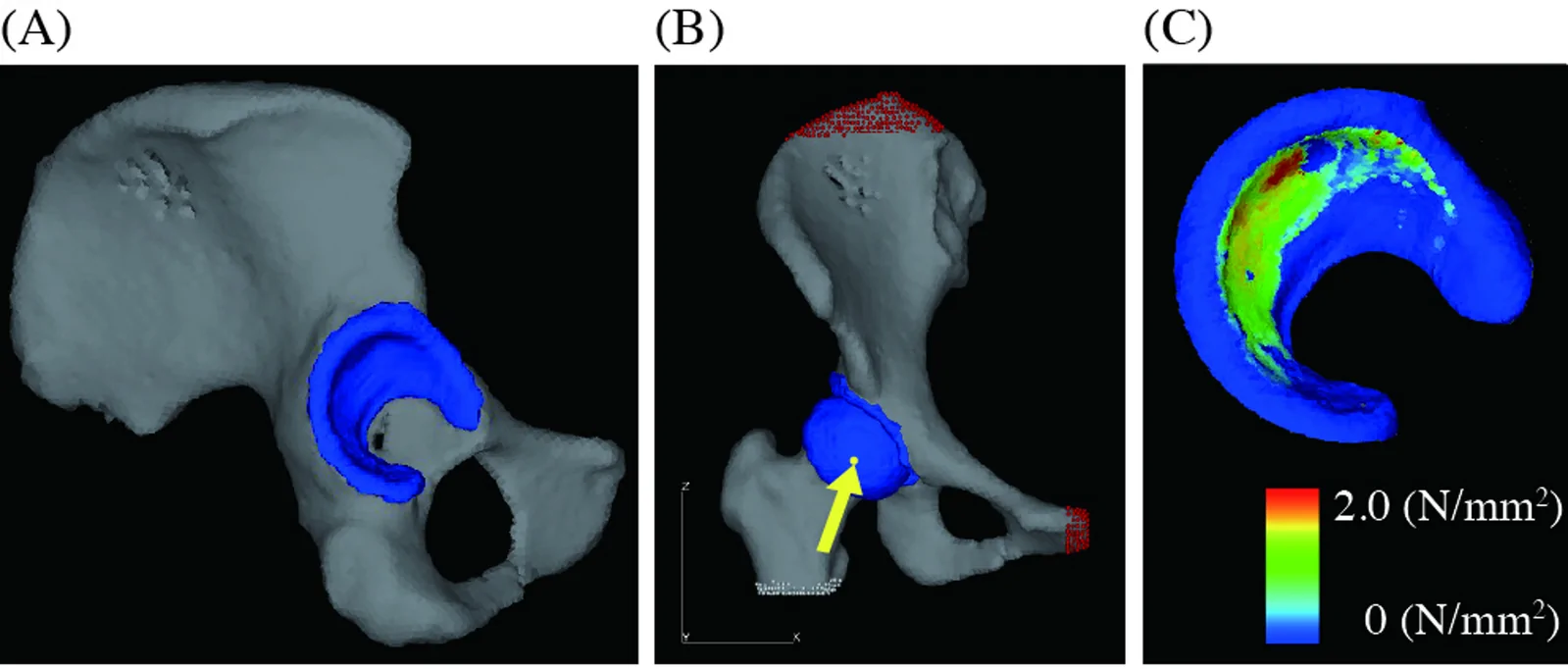
**A** Three-dimensional finite element model of the hemipelvis and acetabular cartilage in a patient with developmental dysplasia of the hip (DDH). The acetabular cartilage was modeled with a uniform thickness of 1.8 mm. **B** In the simulated single-leg standing position, a load is applied at the center of the femoral head. During loading, the iliac crest and pubic region were completely fixed, while the distal femur was constrained in the X- and Y-directions but allowed to move freely in the Z-direction. **C** Distribution of the joint contact pressure on the acetabular cartilage of the right hip in a representative patient with DDH

Three-dimensional finite-element models were generated for each patient using Mechanical Finder software (version 12, Research Center for Computational Mechanics Inc.) based on CT images, with the primary purpose of evaluating the relationship between bony morphology and joint stress. The trabecular and inner cortical bones were modeled using 2 mm tetrahedral elements, while three-nodal point shell elements (0.4 mm thick) represented the outer cortical bone. The acetabular and femoral head cartilages were modeled as homogeneous isotropic materials with a uniform thickness of 1.8 mm (Fig. 3A) [33, 45]. 

A thin shell (0.0005 mm) was applied to the acetabular cartilage surface to evaluate contact pressure distribution. Bone mineral density, derived from CT Hounsfield unit values, was converted into Young’s modulus using the equation proposed by Keyak et al. [3, 17, 21] The Poisson’s ratio of the bone was set to 0.3. The elastic modulus and Poisson’s ratio of the articular cartilage were set to 15 MPa and 0.45, respectively [27, 45]. 

In this model, the cartilage-bone interface was assumed to be fully bonded, whereas the cartilage-cartilage interface allowed frictionless sliding. A loading force of 1158 N was applied at the femoral head center, with body weight standardized at 500 N to prevent scaling effects, following Kitamura et al. [22, 23] The iliac crest and pubic region were completely fixed, while the distal femur was constrained in the X- and Y-directions but allowed free movement in the Z-direction (Fig. 3B). A nonlinear contact analysis was performed to assess the joint contact area and contact pressure on the acetabular cartilage (Fig. 3C).

### Statistical analysis

Linear mixed-effects models with patient ID included as a random effect were used to compare patient demographics, radiological evaluations, finite element analysis evaluations, and HDA percentages between the simple and complicated groups, thereby accounting for within-patient correlations arising from bilateral hips. Linear mixed-effects regression analysis was also conducted to identify the factors influencing HDA percentage. Spearman’s rank correlation analysis was performed to assess associations between radiographic parameters and regional HDA percentages. Chi-square analysis was used to compare the distribution of labral injury types across the three regions. All statistical tests were performed using the SPSS Statistics (IBM Corp., Armonk, NY, USA). Statistical significance was set at *P* < 0.05.

## Results

### Radiological and CT-OAM evaluations

Among the analyzed hips, 43 were classified as Tönnis grade 0 and 11 as Tönnis grade I. All hips were categorized as Crowe classification Type I. The radiological evaluations for the entire study group were as follows: the mean CE angle was 11.1° (95% CI, 8.4–13.9), the mean ARO was 18.4° (95% CI, 16.7–20.2), the mean AHI was 62.4% (95% CI, 59.7–65.1), and mean ACEA was 12.6° (95% CI, 9.3–15.9). Only one hip demonstrated radiographic findings consistent with a femoral cam lesion.

The HDA percentage of the entire study group was calculated using CT-OAM. The mean anterior HDA percentage was 15.3% (95% CI, 12.2–18.4), the mean superior HDA percentage was 68.0% (95% CI, 63.1–72.8), and the mean posterior HDA percentage was 16.6% (95% CI, 13.2–20.0). Spearman’s rank correlation analyses demonstrated a weak positive correlation between the CE angle and posterior HDA percentage (ρ = 0.26, *p* = 0.049), whereas no significant correlations were observed between other radiographic parameters and regional HDA percentages.

### Labral injury distribution

Table 1 summarizes the number of hips with labral injuries categorized according to the anterior, superior, and posterior regions. Anterior labral injuries were observed in all but one hip, with 24 cases classified as complicated. Superior labral injuries were present in 40 hips, including 14 in the complicated group. Posterior labral injuries were identified in 31 hips, with six classified as complicated. Chi-square analysis demonstrated a significant difference in the distribution of labral injury types across the three regions (χ² = 30.7, df = 4, *p* < 0.001). Residual analysis further revealed that anterior–normal and posterior–complicated injuries were significantly less frequent than expected, whereas anterior–complicated and posterior–normal injuries were significantly more frequent than expected (|residual| > 2).

**Table 1:** The number of hips by group for each region

Group	Normal	Simple injury	Complicated injury
Anterior	1	29	24
Superior	14	26	14
Posterior	23	25	6

### Linear mixed-effects regression *to identify the factors influencing HDA percentage*

Linear mixed-effects regression analyses were conducted to assess the factors influencing HDA percentage, with patient ID included as a random effect. Table 2 summarizes the results of the linear mixed-effects regression analyses. The HDA percentages of the three regions (anterior, superior, and posterior) were used as dependent variables. The independent variables included age, the CE angle, ARO, and presence of complicated labral injuries in each region. Complicated injuries were treated as binary categorical variables defined by the presence or absence of complicated injuries in each region. Multicollinearity was evaluated using variance inflation factors (VIFs), and a VIF > 5 was considered indicative of problematic multicollinearity. All variables included in the final model demonstrated VIF values below 2.5 (range, 1.17–2.45), indicating no evidence of problematic multicollinearity.

**Table 2:** Multiple liner regression to predict factors associated with superior HDA percentage

Dependent variable: Superior HDA percentage (R2 = 0.21, F (6,51) = 2.3, *p* = 0.044)				
Independent variable	Estimate	95% CI	T value	P value
Intercept	80.3	35.4–125.1	3.6	< 0.001
CE angle	-0.0	-0.5–0.4	0.2	0.803
ARO	-0.0	-0.9–0.4	0.1	0.846
AHI	-0.2	-0.7–0.3	0.8	0.401
Anterior labral complicated injury	-0.6	-11.2–9.9	0.1	0.898
Superior labral complicated injury	20.5	4.4–36.6	2.5	**0.013**
Posterior labral complicated injury	-2.1	-21.3 − 17.1	0.2	0.825

HDA and high-density area percentages

A significant correlation was found between the superior HDA percentage and the independent variables. Complicated labral injury in the superior region was identified as a positive influencing factor for the superior HDA percentage. In contrast, no significant correlation was observed between the independent variables and the anterior or posterior HDA percentages.

### Comparison of simple and complicated labral injuries

**Fig. 4:**
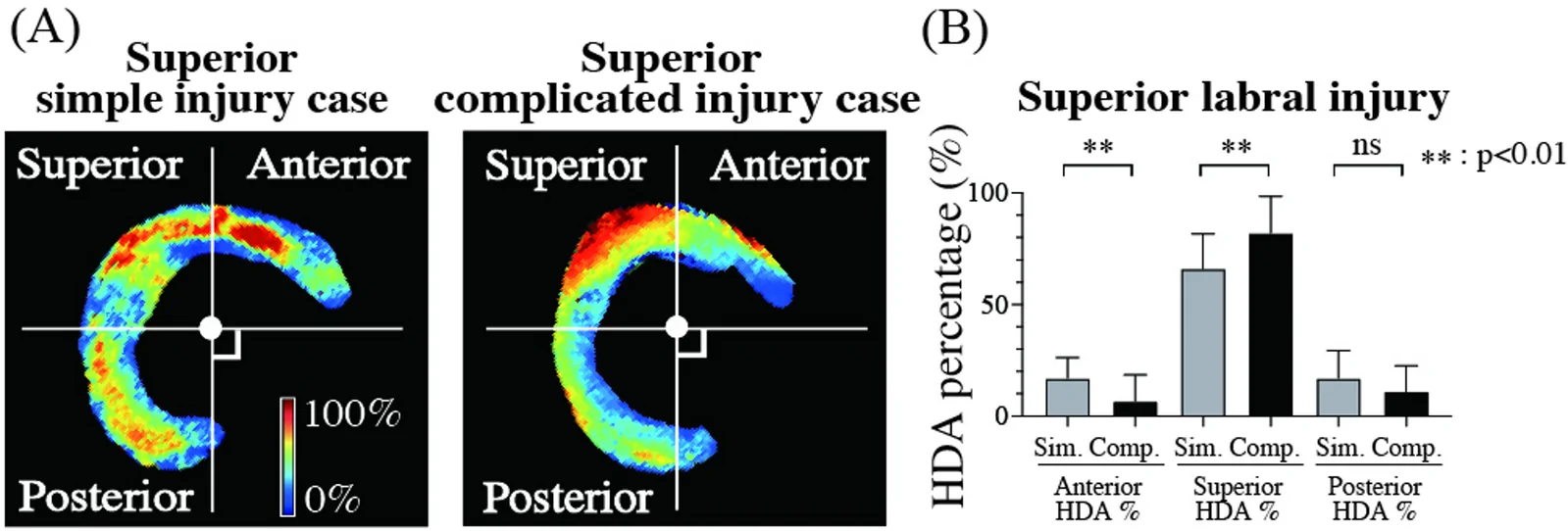
**A** Representative subchondral bone density distributions of simple and complicated labral injuries in the superior region. **B** Comparison of the HDA percentages between the simple and complicated superior labral injury groups. The high-density area (HDA) represented the top 20% of Hounsfield unit (HU) values across the entire articular surface. The HDA percentage was calculated as the proportion of the region occupied by HDA relative to the total area of that region. HDA %, high-density area percentage; sim, simple injury group; comp., complicated injury group

To investigate the characteristics of labral injuries at each site and their impact on joint mechanics, simple and complicated injury groups for each region were compared. For anterior labral injuries, the complicated injury group demonstrated a significantly lower ACEA than the simple injury group (simple vs. complicated: 16.9° (12.6–21.2) vs. 7.7° (2.9–12.4), *p* = 0.004). No significant differences were observed in the other patient demographics or radiological parameters. Regarding HDA percentage distribution, the posterior HDA percentage was significantly lower in the complicated injury group than in the simple injury group (simple vs. complicated: 18.9% (14.5–23.3) vs. 12.6% (7.8–17.4), *p* = 0.033), whereas no significant differences were observed in the anterior or superior HDA percentages. With regard to superior labral injuries, the complicated injury group had a significantly smaller ARO angle than the simple injury group. However, no significant differences were observed in the other radiological parameters. Additionally, the anterior HDA percentage was significantly lower, whereas the superior HDA percentage was significantly higher in the complicated injury group than in the simple injury group (Table 3; Fig. 4). For posterior labral injuries, the complicated injury group demonstrated a significantly lower anterior HDA percentage than the simple injury group (simple vs. complicated: 16.8% (13.6–19.9) vs. 2.9% (− 5.6–11.6), *p* = 0.004). No significant differences were observed in the other radiological parameters or HDA percentages. Additionally, movement-related functional status, as assessed by the JHEQ Movement subscale, did not differ significantly between the groups.

**Table 3:** Comparisons of background of patients, radiological parameters and HDA percentage between the simple and complicated injury groups in superior labral injuries

Superior labral injury	Simple	Complicated	*P* value
Number	29	14	
Age	30.5 (25.2–35.5)	33.3 (26.0–40.7)	0.480
BMI	21.1 (19.7–22.6)	23.6 (21.2–26.0)	0.061
CE angle, degree	10.2 (6.3–14.1)	8.6 (1.0–16.2)	0.670
ARO, degree	20.0 (17.8–22.2)	14.4 (11.5–17.3)	**0.002**
AHI, degree	61.2 (58.3–64.2)	65.4 (57.4–73.3)	0.222
Anterior HDA percentage, %	16.5 (13.0–20.1)	6.9 (0.1–13.6)	**0.005**
Superior HDA percentage, %	65.1 (58.5–71.7)	82.1 (72.7–91.5)	**0.003**
Posterior HDA percentage, %	18.2 (13.1–23.4)	10.9 (4.3–17.6)	0.080

Data are presented as means (95% CI). CE, center edge; AHI, acetabular head index; ARO, acetabular roof obliquity; HDA, high-density area percentage

### FE analysis of loading stress

FE analysis was performed to assess the impact of bone morphology on loading stress differences between the simple and complicated injury groups. Although the anterior complicated injury group exhibited a higher maximum contact pressure than the simple injury group, no significant differences were observed in the maximum contact pressure or contact area between the two groups in the superior region (Table 4). Similarly, no significant differences were observed in the posterior region between the simple and complicated injury groups.

**Table 4:** Comparisons of contact analysis using finite element analysis between the simple and complex injury groups in superior labral injuries

Anterior labral injury	Simple	Complicated	*P* value
Number	29	24	
Maximum contact pressure (N/mm^2^)	3.9 (2.5–5.3)	7.1 (5.3–8.9)	0.005
Contact area, mm^2^	681 (432–930)	481 (324–638)	0.141

Superior labral injury	Simple	Complicated	*P* value
Number	26	14	
Maximum contact pressure (N/mm^2^)	5.2 (3.3–7.2)	6.2 (2.8–9.5)	0.537
Contact area, mm^2^	622 (429–816)	407 (97–717)	0.153

## Discussion

Our study found that complicated injuries to the superior part of the labrum in patients with DDH were associated with localized stress concentration in the hip joint, which is consistent with our hypothesis that the type and location of labral injuries are associated with joint loading stress distribution. In DDH, eccentric loading is known to occur, and radiographic sclerosis is commonly observed in the weight-bearing region. However, how the regional distribution of cumulative loading stress relates to the location and severity of labral injury remains incompletely understood. Using CT-OAM, we observed that patients with complicated superior labral injuries had a significantly higher subchondral bone density in the superior acetabulum, suggesting that joint stress was concentrated in this area. Although the FE analysis did not show a significant difference in the maximum contact pressure between simple and complicated superior labral injuries, this was expected because the FE models incorporated only bony morphology and not the labrum itself. Thus, FE analysis provided a baseline of bone-derived stress, while CT-OAM revealed the cumulative effect of labral pathology. The discrepancy observed between CT-OAM and FEA results therefore does not diminish the validity of our findings, but rather emphasizes that subchondral bone density changes cannot be fully explained by bony morphology alone. Instead, these findings raise the possibility that labral injury may contribute to abnormal load distribution. This interpretation is in line with the technical reality that reliable FE models of the acetabular labrum are not currently feasible due to its complex anisotropic fibrocartilaginous properties. When considered together with cadaveric biomechanical studies demonstrating that labral disruption increases intra-articular stress [4], our findings further support the possibility that superior labral injuries may alter the distribution of joint stresses and contribute to increased intra-articular stress in DDH.

The articular labrum plays a crucial role in hip joint stability by creating a vacuum seal that helps to maintain femoral head containment within the acetabulum. A recent systematic review highlighted the important biomechanical role of the acetabular labrum in maintaining hip stability and load distribution in patients with DDH, supporting the concept that preservation of labral integrity is important for maintaining the mechanical environment of the hip [39]. When the labrum is damaged, this seal is compromised, leading to increased joint instability. In this study, complicated superior labral injuries were associated with changes in the distribution of the HDA percentage, suggesting an altered loading pattern in the superior acetabulum. This observation aligns with cadaveric studies demonstrating that complex labral tears contribute to hip instability [10]. Notably, these changes occur independently of radiological parameters and bone morphology, as finite element analysis did not show significant differences in contact pressure or contact area between the simple and complicated injury groups. Instead, stress concentration was localized at the site of the superior labral injury, suggesting that instability from labral damage may contribute to abnormal stress accumulation. However, whether this relationship reflects a cause or consequence of abnormal loading cannot be determined from the present cross-sectional study.

In this study, labral injuries were most frequently observed in the anterior region, which is consistent with previous reports [12, 26]. Additionally, most complicated labral injuries occurred anteriorly. Anterior labral injuries are common in DDH because mechanical stress is naturally concentrated in the anterior acetabulum, where the joint margin runs parallel to the acetabular rim [5, 42]. Interestingly, despite the increased maximum contact pressure observed in the finite element analysis for the complicated injury group, there was no significant difference in the anterior HDA percentage between the simple and complicated anterior injury groups. This finding suggests that, unlike the superior region, the anterior labrum in DDH may be inherently predisposed to damage owing to its structural and mechanical loading characteristics rather than progressive stress accumulation alone. In other words, because mechanical stress is already concentrated in the anterior acetabulum in DDH, anterior labral injuries may occur without being accompanied by additional changes in subchondral bone density.

To the best of our knowledge, no finite element models have been established that can directly compare the severity of labral injuries, such as simple versus complex tears, and constructing such models was not feasible in this study. Accordingly, our finite element model focused on bony morphology and articular cartilage and was used to evaluate the influence of bone morphology on joint stress. The discrepancy observed between CT-OAM and FEA results may partly reflect the effect of bony morphology on the stress environment. However, this factor alone does not fully account for the differences, and the contribution of additional factors should be considered. Notably, FEA in this study evaluated instantaneous stresses under a single-leg stance, whereas CT-OAM reflects the cumulative history of repetitive loading during daily activities, suggesting the additional influence of dynamic movements. Moreover, the acetabular labrum is richly innervated, and labral injury has been reported to alter neuromuscular control, thereby affecting movement patterns [7]. Experimental evidence also indicates that the magnitude of labral strain varies depending on injury location during hip motion, suggesting that the site of labral injury may differentially influence joint mechanics during dynamic activities [38]. Taken together, these findings suggest that labral injuries may not only passively reflect abnormal joint loading but may also actively contribute to altered stress distribution by modifying movement patterns, underscoring the need for future studies incorporating motion analysis.

This study had several limitations. First, the stress distribution in the hip joint was not measured directly. Instead, we evaluated the distribution pattern of subchondral bone density using the CT-OAM method, which may not fully reflect absolute stress values. In addition, the presence of subchondral bone cysts can potentially affect the measurement values. However, this study included only patients with DDH who had no acetabular bone cysts because they were candidates for joint preservation surgery. Second, the study population consisted of patients who underwent joint-preserving surgery for DDH, primarily those with symptoms such as pain. This selection may have led to an overestimation of the frequency and severity of labral injuries compared to the broader DDH population, and the findings may not be generalizable to less severe cases that do not require surgical intervention. In addition, intact labra were extremely rare in this cohort, making comparisons between hips with and without labral injuries difficult. Nevertheless, focusing on this symptomatic group allowed for a clearer identification of site-specific risks for labral injury. Third, as a cross-sectional study, this analysis did not account for changes in labral damage or joint loading stress over time. Although longitudinal studies are needed to clarify the long-term effects, the combined use of CT-OAM and finite element analysis provides valuable insights into the relationship between labral injury and stress distribution. Furthermore, a uniform body weight was assumed for all participants. This approach does not account for the effect of individual body weight on contact pressure, but was adopted to eliminate the scaling effect of body weight on absolute pressure values and to focus on the influence of morphological differences on the stress environment. Several subgroup analyses included relatively small numbers of patients. Therefore, the absence of significant differences in finite element analysis parameters should be interpreted with caution because of the potential for Type II error. Finally, the study included only female patients, reflecting the higher prevalence of DDH in women; therefore, the associations observed in this study may not necessarily apply to male patients.

## Conclusion

This study demonstrated that complicated superior labral injuries in female patients with DDH are associated with localized stress concentrations in the superior acetabulum. CT-OAM revealed that the severity of superior labral injury correlates with a characteristic HDA distribution pattern in subchondral bone density, which reflects cumulative loading. These loading patterns were not fully explained by finite element analysis based on bony morphology alone. Furthermore, anterior labral injuries were the most frequent; however, their occurrence may be influenced more by the inherent joint structure than by progressive stress accumulation. A better understanding of these relationships may help refine treatment strategies for DDH by considering the severity of the labral injury and its impact on joint mechanics.

## Data Availability

No datasets were generated or analysed during the current study.
